# Biochemical, Histological, and Multi-Omics Analyses Reveal the Molecular and Metabolic Mechanisms of Cold Stress Response in the Chinese Soft-Shelled Turtle (*Pelodiscus sinensis*)

**DOI:** 10.3390/biology14010055

**Published:** 2025-01-11

**Authors:** Liqin Ji, Qing Shi, Chen Chen, Xiaoli Liu, Junxian Zhu, Xiaoyou Hong, Chengqing Wei, Xinping Zhu, Wei Li

**Affiliations:** Key Laboratory of Tropical and Subtropical Fishery Resources Application and Cultivation, Ministry of Agriculture and Rural Affairs, Pearl River Fisheries Research Institute, Chinese Academy of Fishery Sciences, Guangzhou 510380, China; jiliqin@prfri.ac.cn (L.J.);

**Keywords:** Chinese soft-shelled turtle, cold stress, apoptosis, morphology, transcriptome, metabolism, signaling pathways

## Abstract

The Chinese soft-shelled turtle (*Pelodiscus sinensis*), a type of warm-water reptile, is frequently chosen as the model animal to understand how organisms respond to environmental stressors. However, the responsive mechanism of *P. sinensis* to natural cold stress is unclear, especially in terms of metabolic pattern and molecular pathways. Herein, plasma biochemical, hepatic morphological, apoptotic, transcriptomic, and metabolomic detection methods were performed to investigate the response of *P. sinensis* to acute cold stress. A consistent increase in plasma AST and ALT activities with a decline in ALP activity was found following 14 °C and 7 °C cold stress compared with the control group. Plasma GLU, TG, CHO, and HDL contents, reflecting energy metabolism, were decreased to lower levels from 2 to 16 days post cold stress (dps). Histological and TUNEL detection in the liver demonstrated that the 14 °C and 7 °C cold stress caused severe morphological damage and cell apoptosis in a time-dependent manner. DEGs in the biosynthesis of fatty acids (*Acsbg2, Acsl3, Acsl4, Acsl5, Mcat,* and *Acacb*), as well as unsaturated fatty acids (*Hsd17b12*, *Elovl7*, *Scd*, and *Baat*), starch and sucrose metabolism (*Pgm1*, *Pgm2*, and *Treh*), and apoptosis (*Ddit3*, *Gadd45a*, *Lmnb1*, *Tuba1c*, *Tnf*, *Tnfsf10*, *Fos*, *Itpr1*, and *Ctso*) were discovered in the transcriptome under cold stress. The metabolomic data showed that metabolites, including chenodeoxycholic acid, oleoylethanolamide, uric acid, fructose 1,6-bisphosphate, CMP, and S-(Hydroxymethyl)-glutathione, were remarkably altered in the cold stress groups. Combined transcriptomic and metabolomic data revealed that pyrimidine metabolism, amino acid metabolism, and pyruvate metabolism were the most significant pathways regulated by the low-temperature exposure. Overall, this work suggests that 14 °C and 7 °C cold stress could induce obvious morphological damage and apoptosis in the liver at 4 dps. Moreover, energy metabolism and amino acid metabolism were the main signaling pathways in response to cold stress for *P. sinensis*.

## Simple Summary

As a warm-water species, The Chinese soft-shelled turtle is fragile to external cold stress. It has been reported that the sudden temperature decline can lead to massive turtle death, which brings about huge economic loss in the farming industry. Our study can help us to understand how cold stress can damage turtles. In addition, it can promote knowing about the responding mechanism of turtles to low temperatures with molecular, metabolic, and pathway analyses.

## 1. Introduction

The continuous release of greenhouse gases into the Earth’s atmosphere by humans is a direct cause of global warming [1]. With global warming, the higher temperature in the surface air brings about extreme weather events that are increasingly intense and frequent [2]. Since 1990 up to recent years, this extreme weather has represented an increase in cold air outbreaks and/or heavy snowfalls across the Northern Hemisphere [3,4]. For example, recent extreme winters have been reported in Europe, the United States [5], and Asia [6].

Extreme cold waves caused by extreme weather events are uncommon, unpredictable, and often short. They are found to not only have a great impact on human health [1], but also have pernicious effects on the wild teleost and aquaculture industry [7]. Extremely low temperature can significantly alter water’s physical and chemical properties, such as permeability, stratification, anaerobic bacteria proliferation, and algae death [7]. Furthermore, it can directly influence the growth, behavior, physiology, and reproduction of ectotherms. Frequent and long durations of cold stress can even lead to increasing mortality and economic losses of aquatic species [8]. Many studies have reported the effect of cold stress on various aquatic animals, such as largemouth bass [9], tilapia [10], and sea turtles [11].

When the ambient temperature decreases in a moderate range, the animals can develop multiple physiological strategies to cope with cold stress, such as the initiation of antioxidant defense, regulation of membrane fluidity, and maintenance of energy homeostasis [12]. For example, cold stress induces organ damage mainly via the oxidative stress caused by the remarkable accumulation of reactive oxygen species (ROS) in fishes [9,13] and mammals [14]. In addition, cold stress can affect the balance of energy metabolism and depress immune capacity in fish [15], which then alters metabolites, such as triglycerides and glucose [16], as well as immune-related parameters, such as alkaline phosphatase (ALP) and acid phosphatase [12].

As a vital organ involved in maintaining metabolic homeostasis and immune tolerance, the liver is sensitive to various physiological stressors. Persistent or intensive stress can lead to substantial damage to the hepatocytes, generally presented as abnormalities in histomorphology and biochemistry parameters [17,18]. Therefore, revealing the metabolic and molecular mechanisms of the liver under low temperature can promote a comprehensive understanding of the adaptive strategies of aquatic animals to cold weather.

In recent years, the combination of transcriptome and metabolome analyses has been widely applied to study the underlying mechanisms of aquatic animals in resisting various stressors, such as cold stress in black porgy (*Acanthopagrus schlegelii*) [13], heat stress in largemouth bass (*Micropterus salmoides*) [9], and hypoxic stress in darkbarbel catfish (*Pelteobagrus vachelli*) [19]. In addition to the analysis of functional molecular mechanisms and metabolites, the related pathways involved in the stress response can also be found by multi-omics techniques. However, the application of multi-omics techniques in studying the effect of cold stress on turtles is rarely reported, which has hampered the recognition of the mechanism of responding to cold stress in reptiles.

Turtles, as a representative group of reptiles, are widely distributed across freshwater as well as the oceans and play a crucial role in aquatic ecosystems [20]. As a freshwater turtle, the Chinese soft-shelled turtle is a naturally subtropical and temperate species, and now is a popular economic aquaculture species owing to its nutritional and medical values in Southeast Asia, including China and Japan. In China, the annual production of the Chinese soft-shelled turtle rose to 497,536 tons in 2023 [21]. When the outdoor water temperature decreases to 4 °C in winter, the Chinese soft-shelled turtle gradually enters into hibernation, which can be beneficial to avoid body freezing and overcome damage. However, prior to hibernation, the outdoor turtles generally experience a period of acute temperature decrease in the spring and autumn, approximately from the end of October or November to March or April of the following year, when the weather is usually changeable, with temperature decreasing by 10–20 °C in one day [22]. This sharp temperature decrease is deemed to cause a large quantity of Chinese soft-shelled turtles to die, which leads to substantial economic losses in the cultural industry [23]. However, the damage mechanism of Chinese soft-shelled turtles caused by acute cold stress remains unclear.

This research comprehensively integrates biochemical and histological techniques with transcriptome and metabolome analysis to reveal the influence of cold stress on the Chinese soft-shelled turtle. This study further describes the regulation mechanism of turtles under acute cold stress and provides a scientific basis for the breeding of cold-resistant Chinese soft-shelled turtles for aquaculture.

## 2. Materials and Methods

### 2.1. Experimental Animals and Design

All experimental procedures and animal treatments were carried out obeying the guidelines for the care and use of laboratory animals in China. This experiment was approved by the Ethics Committee of the Pearl River Fisheries Research Institute, Chinese Academy of Fishery Sciences (LAEC-PRFRI-2023-10-15).

A total of 600 juvenile Chinese soft-shelled turtles with an average body weight of 12 ± 3 g were kindly gifted by Huizhou Wealth Xing Industrial Co., Ltd. (Huizhou, China) and transported to the Guangzhou Aquatic Thoroughbred Base of the Pearl River Fisheries Research Institute (Guangzhou, China), where the experiment was performed. Afterwards, the animals were acclimated in 2 acrylic tanks (1 m × 1 m × 0.25 m) and fed with commercial pellet diets kindly gifted by Guangdong Nutriera Group Co., Ltd. (Guangzhou, China), with product number 0081. During the 14-day acclimation period, the aerated water parameters were as follows: temperature 28 ± 1 °C, pH 8.2 ± 0.4, NH_4_-N 4.1 ± 1.1 mg/L, NO_2_^-^ 1.0 ± 0.3 mg/L, and dissolved oxygen 5.8 ± 1.5 mg/L. The turtles were fed twice a day at 9:00 and 16:00 until apparent satiation. One-third of the rearing water was exchanged once a week during acclimation.

**Figure 1:**
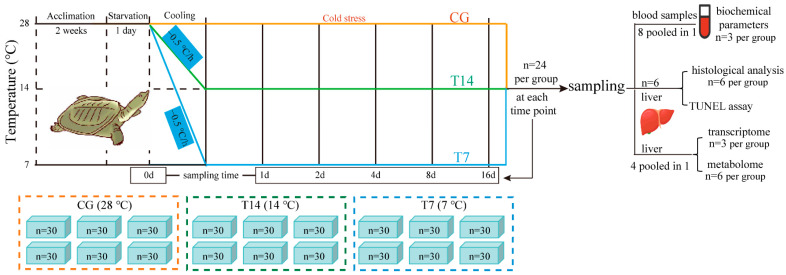
Experimental flow chart.

As shown in Figure 1, the 540 healthy turtles were randomly transferred to 18 small acrylic boxes (30 individuals per box) of a size of 37 cm × 25 cm × 11 cm after acclimation. Then, these turtles were starved for 24 h for the subsequent cold stress experiment. Three groups were set in the cold stress experiment, separately defined as the 28 °C control group (CG), 14 °C cold stress group (T14), and 7 °C cold stress group (T7). Each group was made up of six boxes containing 180 turtles (30 individuals per box). In the CG, the water temperature was maintained at 28 °C throughout the experiment, which is optimal for the survival and growth of turtles. In the T14 and T7 groups, the water temperature was decreased from 28 °C to 14 °C and 7 °C at a rate of 1 °C every 2 h using RXZ-436 cooling incubators (Ningbo Jiangnan Instrument Factory, Ningbo, China). After the cooling process, the water temperature was constantly kept at 28 °C, 14 °C, and 7 °C, respectively, in the CG, T14, and T7 groups until the end of the cold stress experiment. During the cold stress, the turtles in the T14 and T7 groups hardly ate food, which is consistent with the natural reaction of turtles to low ambient temperature. To avoid the effect of food intake on the experimental results, food was withheld from the turtles in all three groups throughout the cold stress experiment.

### 2.2. Sample Collection

The time point decreasing to the designated temperature was defined as the onset of the cold stress. The individuals in the three groups were sampled before cooling stress, and at 1, 2, 4, 8, and 16 days post cold stress (dps) (Figure 1). A total of 24 biological replicates per group (4 individuals per box) were sampled at each time point (n = 24). All turtles were anesthetized with 1 g/L tricaine methanesulfonate (MS-222) solution prior to obtaining blood samples from the neck–chest fracture section. The blood samples were collected for subsequent plasma biochemical analysis. Afterwards, the turtles were dissected rapidly to collect the liver tissue. A part of the liver was fixed in Bouin’s solution overnight for histological staining and a TUNEL assay. The remaining livers were snap-frozen in liquid nitrogen and stored at −80 °C for transcriptome and metabolome detection.

### 2.3. Plasma Parameters

To reduce individual differences, blood samples from 8 individuals were mixed into one 5 mL vacutainer with heparin sodium (n = 3 per group). After being kept at 4 °C for 5 h, the blood samples were centrifuged at 4000× *g* for 20 min at 4 °C to obtain supernatant plasma, which was stored at −80 °C for the detection of biochemical parameters.

The contents of plasma glucose (GLU, kit number: A154-1-1), triglyceride (TG, A110-1-1), total cholesterol (CHO, A111-1-1), high-density lipoprotein cholesterol (HDL, A112-1-1), and low-density lipoprotein cholesterol (LDL, A113-1-1), as well as the activities of plasma aspartate aminotransferase (AST, C010-1-1), alanine aminotransferase (ALT, C009-3-1), and ALP (A059-1-1), were detected with commercial assay kits following the manufacturer’s instructions (Nanjing Jiancheng Bioengineering Institute, Nanjing, China). For every group, we detected three pooled repeats for each parameter at each time point (n = 3).

### 2.4. Histological Analysis and TUNEL Assay

Six replicates of livers per group were collected for histological detection (n = 6). The collected liver tissues (smaller than 1 cm × 1 cm × 1 cm) were rapidly immersed in Bouin’s solution at room temperature overnight. After 24 h of fixation, the liver tissues were washed 2–3 times with 70% ethanol and preserved in 70% ethanol. The samples were subsequently dehydrated with alcohol gradients, embedded in paraffin, sliced to 4 μm thickness, and stained with H&E (hematoxylin and eosin). The slices were sealed with resin and observed with a Nikon ECLIPSE C1 optical microscope (Tokyo, Japan). The counts of hemosiderin deposition, inflammatory cell infiltration, and hepatocytes with steatosis were measured in a randomly chosen 0.01 mm^2^ field. The amounts of the three parameters are exhibited as counts per mm^2^ in figures.

To detect cell apoptosis, TUNEL staining was carried out with the one-step TUNEL apoptosis assay kit purchased from Wuhan Servicebio Technology Co., Ltd. (#G1504, Wuhan, China). The immunofluorescence images were visualized with the Nikon Eclipse Ti confocal laser scanning microscope (Tokyo, Japan) with the Pannoramic MIDI imaging system (Budapest, Hungary). The percentage of apoptotic cells was calculated by the ratio of the number of TUNEL-positive cells relative to the total number of DAPI-labeled nuclei in five non-overlapping visual fields with ImageJ2 software [24].

### 2.5. Transcriptomic Analysis of Liver Samples

Livers of 24 individuals per group were sampled with 4 individual livers equally mixing into 1 sample, which meant each group had 6 pooled samples at each time point. Three pooled samples of each group (n = 3) were randomly chosen to extract the total RNA with the TRIzol reagent (Invitrogen Life Technologies, Carlsbad, CA, USA). The liver RNA of the CG, as well as the T14 and T7 groups at 8 dps, was paired-end sequenced on the NovaSeq 6000 platform (Illumina, San Diego, CA, USA) by Suzhou PANOMIX Biomedical Tech Co., Ltd. (Suzhou, China) after assessing RNA quality and constructing the library. The clean data screened from the raw data after removing the adapter and low-quality reads were mapped to the genome of *P. sinensis* (https://www.ncbi.nlm.nih.gov/datasets/genome/GCF_000230535.1/) accessed on 24 July 2012.

The gene expression levels were calculated as FPKM (fragments per kilobase per million mapped fragments). The differentially expressed (DEGs) genes between three comparisons (CG vs. T14, CG vs. T7, and T14 vs. T7) were identified with a ∣log_2_ (fold change)∣ ≥ 1.0 and adjusted *p*-value < 0.05. The DEGs’ functions were annotated with the gene ontology (GO) and KEGG (Kyoto Encyclopedia of Genes and Genomes) public databases.

### 2.6. Metabolomic Analysis of Liver Samples

Six pooled samples per group (n = 6) at 8 dps were selected for metabolomic analysis for the three groups. The 50 mg pooled liver tissue was homogenized, processed, and centrifuged to obtain the supernatant. The 60 µL supernatant was detected via liquid chromatography tandem–mass spectrometry system (LC-MS), guided by the previous method [25]. The metabolome analysis was carried out by Suzhou Panomix Biomedical Tech. Co., Ltd. (Suzhou, China). Ultra-high-performance liquid chromatography (UHPLC) was performed with the Thermo Vanquish system (Thermo Fisher Scientific, Waltham, MA, USA). Quality control (QC) samples, equal to the number of experimental samples, were used to determine the stability and reliability of the system. Mass spectrometry was performed on the Thermo Q Exactive HF-X mass spectrometer (Thermo Fisher Scientific, Waltham, MA, USA) in positive- and negative-polarity modes.

The raw data, following conversion into mzXML format, were analyzed to describe the peak identification, peak filtration, and peak alignment. The metabolites were annotated using public metabolites databases, including HMDB (http://www.hmdb.ca/), Metlin (http://metlin.scripps.edu/index.php, accessed on 24 July 2012), Massbank (http://www.massbank.jp/), LIPID MAPS (https://lipidmaps.org/), mzCLOUD (https://www.mzcloud.org/), and the BioNovoGene database (http://www.bionovogene.com). The R language package was executed to accomplish the multivariate statistical analysis of the metabolites, including partial least squares discriminant analysis (PLS-DA) and orthogonal partial least squares discriminant analysis (OPLS-DA). Projections of important variables (VIPs) > 1 and *p* < 0.05 were set to screen the differentially expressed metabolites (DEMs) between groups. The DEMs were functionally annotated with the KEGG database.

### 2.7. Joint Analysis of Transcriptome and Metabolome

DEGs (FDR < 0.05 and |log2FC| > 1) and DEMs (VIP > 1 and *p* < 0.05) were integrated for the pairwise comparisons. The Pearson model was established to evaluate the correlation between the DEGs and DEMs using the Pearson correlation coefficient (PCC) and the relevant *p* value. Those with |PCC| > 0.80 and *p* < 0.05 were considered to be significantly correlated. The DEGs and DEMs were further mapped to the KEGG database to obtain their shared pathways. Finally, the network of the DEGs and DEMs was established to reveal the signaling pathways regulated by low temperatures.

### 2.8. Validation of Transcriptomic Results by qRT-PCR

The reliability of the transcriptomic data was validated by quantitative real-time PCR (qRT-PCR). The liver tissues used in the qRT-PCR validation were the same as those in the transcriptome. The total liver RNA of the CG and the T14 and T7 groups at 8 dps was extracted with TRIzol Reagent (Roche, Belmont, CA, USA). The purified RNA was reversely transcribed into cDNA following the digestion of genomic DNA from the RNA. The reaction system of qRT-PCR included 2 μL cDNA (~1 μg), 10 μL of 2 × SYBR Green Master Mix (Takara, Dalian, Chian), 4 μM of each primer, 0.4 μL of ROX reference dye, and RNase-free water to a final volume of 20 μL. The reaction system was run in the ABI StepOnePlus System (Applied Biosystems, Foster, CA, USA) by setting the program as follows: 95 °C for 5 min, followed by 40 cycles of 95 °C for 5 s and 60 °C for 30 s. Each sample was run in triplicate for technical repeats. Ten DEGs were randomly selected for validation, with *β-actin* chosen as the reference gene. The primers used in the qRT-PCR are shown in Table S1. The 2^−△△CT^ method was applied to determine the fold changes of the mRNA levels in the treatment group relative to the control group [26].

### 2.9. Statistical Analysis

All data are shown as mean ± standard error (SE) or standard deviation (SD). The plasma biochemical, histopathological, and cell apoptosis results of the three groups at the same time points were statistically analyzed by one-way analysis of variance (ANOVA) followed by Duncan’s post hoc test. *p* < 0.05 was considered to be statistically significant. The correlation between the transcriptomic results and qRT-PCR results was evaluated by Pearson R^2^ values and plotted with GraphPad Prism 9.0 software. The IBM SPSS 21.0 software (Armonk, New York, NY, USA) was utilized for statistical analysis.

## 3. Results

### 3.1. Plasma Biochemical Parameters During Cold Stress

**Figure 2:**
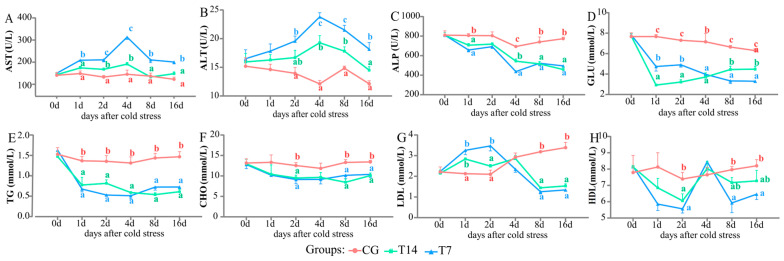
Effects of cold stress on plasma biochemical parameters (**A**–**H**). (**A**) Aspartate aminotransferase (AST) activity, (**B**) alanine aminotransferase (ALT) activity, (**C**) alkaline phosphatase (ALP) activity, (**D**) glucose (GLU) content, (**E**) triglyceride (TG) content, (**F**) total cholesterol (CHO) content, (**G**) low-density lipoprotein cholesterol (LDL) content, and (**H**) high-density lipoprotein cholesterol (HDL) content. All data are represented as mean ± SE (n = 3). Different superscript letters mean significant differences in different groups at the same time point (*p* < 0.05).

The activities of plasma AST, ALT, and ALP can be used to evaluate tissue health; meanwhile, the contents of plasma GLU, TG, CHO, HDL, and LDL can reflect energy metabolism (Figure 2A–H). Compared with the CG, the activities of AST (Figure 2A) and ALT (Figure 2B) were increased at 2 dps and reached the highest level at 4 dps in the T7 and T14 groups (*p* < 0.05). They were consistently higher in the T7 group than that in the T14 group and CG from 2 to 16 dps (*p* < 0.05). ALP activity (Figure 2C) was kept at lower levels in the T14 and T7 groups compared with the CG from 1 to 16 dps; moreover, ALP activity in the T7 group reached the lowest at 4 dps (*p* < 0.05). In the T14 and T7 groups, the contents of GLU (Figure 2D), TG (Figure 2E), and CHO (Figure 2F) were all significantly decreased to lower levels in comparison with the CG from 2 to 16 dps (*p* < 0.05). The GLU content in the T7 group was higher than that in the T14 group at 1 and 2 dps; however, this trend was reversed at 8 and 16 dps (*p* < 0.05). No significant difference was found in the TG contents between the T7 and T14 groups during the cooling process (*p* > 0.05), and CHO acted in the same way. The LDL content (Figure 2G) increased to higher levels in the T14 and T7 groups at 1 and 2 dps, while it decreased to lower levels from 8 to 16 dps in the T14 and T7 groups compared with the CG (*p* < 0.05). In contrast with LDL, HDL (Figure 2H) content in the T7 group significantly declined at 2, 8, and 16 dps compared with the CG (*p* < 0.05). The variation in HDL content in the T14 group showed a similar trend to that in the T7 group.

### 3.2. Cold Stress Induced the Damage of Hepatic Morphology

**Figure 3:**
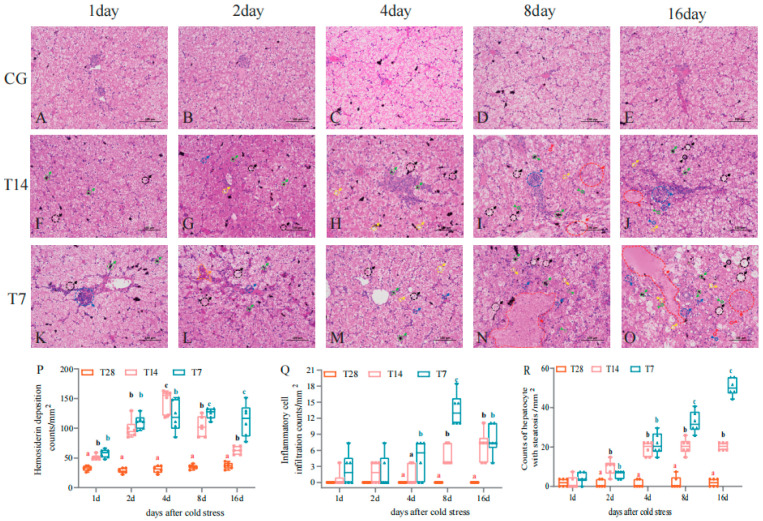
The representative histopathological traits of Chinese soft-shelled turtle liver at different time points post cold stress. (**A**–**E**) CG (28 °C control group). (**F**–**J**) T14 (14 °C cold stress group). (**K**–**O**) T7 (7 °C cold stress group). (**P**–**R**) Quantitative analysis of the counts of hemosiderin deposition (**P**), inflammatory cell infiltration (**Q**), and hepatocytes with steatosis (**R**). Scale bar = 100 μm. The orange arrows indicate that hepatic sinuses were enlarged and congested with erythrocytes. The red, blue, black, yellow, and green arrows, respectively, represent cell necrosis, inflammatory cell infiltration, cell edema, steatosis, and hemosiderin deposition. The blue, black, green, and red triangles, respectively, point to hepatocyte necrosis with acidophilic bodies, nuclear migration, nuclear deformation, and nuclear swelling. The quantitative data are represented as mean ± SD (n = 6). Different letters mean significant differences in different groups at the same time point (*p* < 0.05).

As shown in Figure 3, the 14 °C and 7 °C cold stress induced histopathological changes in the liver, detected by H&E staining. In the CG (Figure 3A–E), hepatocytes with normal round nuclei were tightly arranged, exhibiting a clear and complete hepatic cord, as well as narrow hepatic sinuses at all time points. In the T14 and T7 groups, the hepatocyte boundary was distinct from 1 (Figure 3F,K) to 2dps (Figure 3G,L), and became obscure at 4 dps (Figure 3H,M) and severely dissolved at 8 (Figure 3I,N) and 16 dps (Figure 3J,O). The hepatic sinuses were enlarged and congested with erythrocytes at 2 dps in the T7 group (Figure 3L, orange arrows). Various kinds of injuries were found inside the cells in the T14 and T7 groups, including hepatocyte necrosis with acidophilic bodies (Figure 3J,O, blue triangle), edema (Figure 3I, black arrows), inflammatory cell infiltration perimysial vessels (Figure 3J, blue arrows), steatosis (Figure 3H, yellow arrows), increased hemosiderin deposition (Figure 3G, green arrows), nuclear migration (Figure 3J,O, black triangle), nuclear deformation (Figure 3J,O, green triangle), and nuclear swelling (Figure 3O, red triangle). In addition, large-scale hepatocyte necrosis (Figure 3N,O, red arrows) was observed from 8 to 16 dps in the T7 group. Statistical analysis was performed on the counts of three kinds of injuries (Figure 3P,R), consisting of hemosiderin deposition (green arrows), inflammatory cell infiltration (blue arrows), and steatosis (yellow arrows). The amounts of hemosiderin deposition were significantly increased in the T14 and T7 groups compared with the CG from 1 to 16 dps (*p* < 0.05), reaching the largest volume in the T14 and T7 groups at 4 dps (*p* < 0.05) (Figure 3P). The counts of inflammatory cell infiltration in the T14 and T7 groups were greater than those in the CG from 8 to 16 dps (*p* < 0.05); moreover, the amounts in the T7 group were higher than the other two groups from 4 to 8 dps (*p* < 0.05) (Figure 3Q). The number of hepatocytes with the steatosis was obviously increased in the T14 and T7 groups compared with the CG from 2 to 16 dps (*p* < 0.05), with the highest number in the T7 group from 8 to 16 dps (*p* < 0.05) (Figure 3R). In general, the 14 °C and 7 °C low temperatures induced multiple types of liver injuries, which were severe at the later stage from 4 to 16 dps. Moreover, the liver damage in the T7 group was intensified in comparison with the T14 group.

### 3.3. Cold Stress Intensified Cell Apoptosis

**Figure 4:**
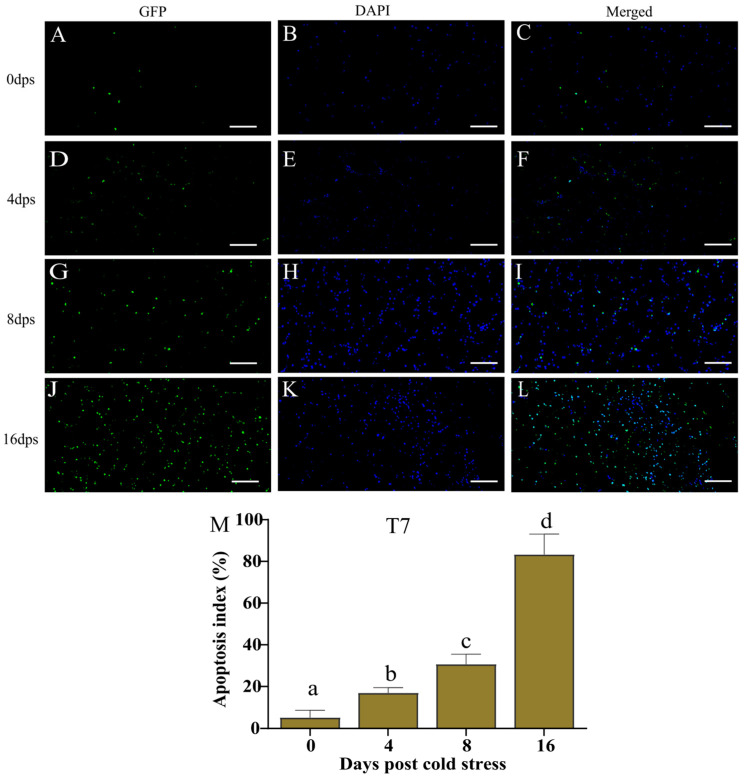
TUNEL staining of liver in the Chinese soft-shelled turtle at different time points post 7 °C cold stress. (**A**,**D**,**G**,**J**) TUNEL-labeled cells (GFP). (**B**,**E**,**H**,**K**) DAPI-labeled nuclei (blue). (**C**,**F**,**I**,**L**) DAPI-TUNEL double-labeled cells (merge). Scale bar = 50 μm. dps indicates days post cold stress. (**M**) Percentage of cell apoptosis. Different letters describe significant differences among groups (*p* < 0.05).

The histopathological results showed that the hepatocyte injuries in the T7 group were more severe than those in the T14 group. Herein, the liver tissues in the T7 group at different time points are chosen to exhibit the effect of cold stress on hepatocyte apoptosis (Figure 4A–L). The images (Figure 4M) show that only 5.21% of GFP-labeled apoptotic cells were detected at 0 dps in the T7 group, which increased to 16.89% at 4 dps (*p* < 0.05). The apoptotic percentage was greatly elevated to 30.69% and 83.3%, respectively, at 8 and 16 dps, significantly higher than at 0 and 4 dps (*p* < 0.05). In general, hepatocyte apoptosis was gradually aggravated from 4 to 16 days after the 7 °C cold stress.

### 3.4. Effect of Cold Stress on Hepatic Transcriptome

The transcriptomic data showed that 44075707, 42862285, and 44157229 raw reads were sequenced in the CG, T14, and T7 RNA-Seq libraries (Table S2). After removing the adaptors and low-quality sequences, a total of 43471642, 42293299, and 43574465 clean reads were obtained for the CG and T14 and T7 groups, which contained 50.25%, 50.27%, and 50.25% CG content, respectively. The average map ratios of genes matching with the Chinese soft-shelled turtle’s genome were all over 88% in the three groups. These data suggest the high quality of the transcriptomic sequencing data.

**Figure 5:**
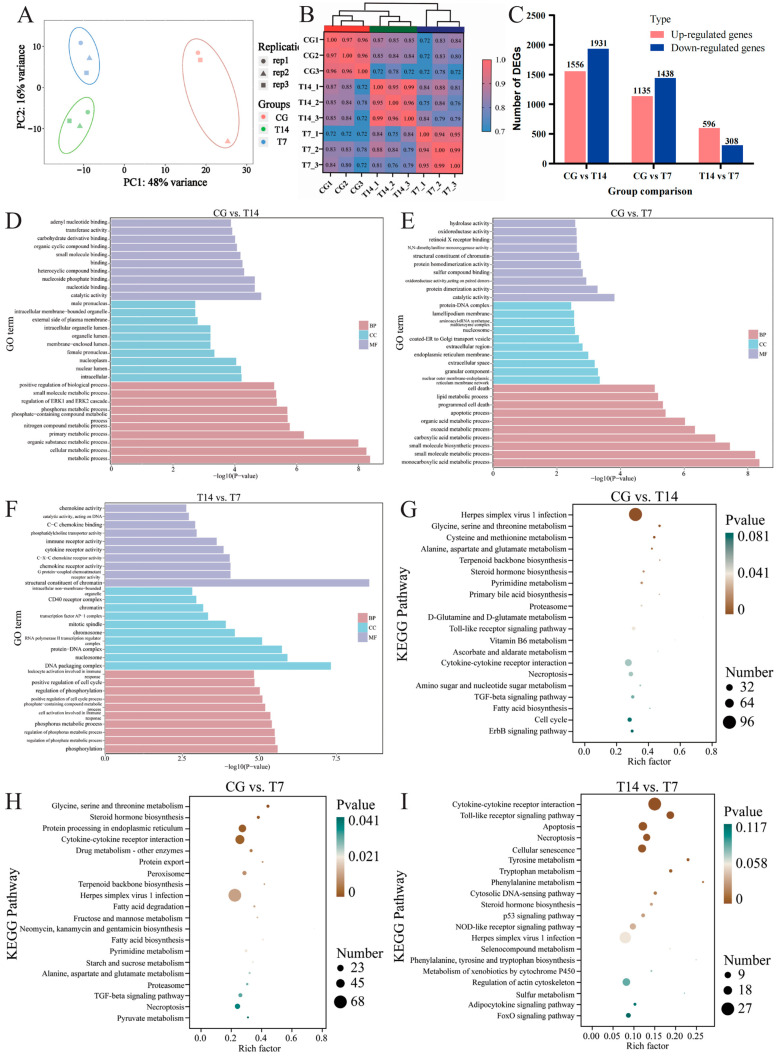
Characterization of transcriptomic profiles in three groups. (**A**) Principal component analysis (PCA) based on all expressed genes in three groups. (**B**) The correlation heatmap of all expressed genes in three groups. (**C**) The number of differentially expressed genes (DEGs) filtered by |log2(FoldChange)| > 1 and adjusted *p*-value < 0.05 in CG vs. T14, CG vs. T7, and T14 vs. T7 comparisons. Red and blue colors, respectively, represent up- and down-regulated genes. GO (**D**–**F**) and KEGG (**G**–**I**) enrichment analyses of DEGs in three pairwise comparisons. “CG” indicates the control group. “T14” and “T7” indicate 14 °C and 7 °C cold stress groups.

PCA can reveal the difference in gene expression patterns in different groups. Our PCA data (Figure 5A) found that the first principal component (PC1) and the second principal component (PC2), respectively, accounted for 48% and 16% of the total variation. This indicated that PC1 was the predominant component in separating the three groups. The correlation heatmap (Figure 5B) showed that the correlation coefficients of the three biological replicates in each group were all larger than 0.9, indicating a high correlation of biological replicates within the group. A total of 3487 (1556 up-regulated and 1931 down-regulated), 2573 (1135 up-regulated and 1438 down-regulated), and 904 (596 up-regulated and 308 down-regulated) DEGs were separately obtained in the CG vs. T14, CG vs. T7, and T14 vs. T7 comparisons (Figure 5C).

Subsequently, GO (Figure 5D–F) and KEGG (Figure 5G–I) enrichment analyses were performed to identify the functional classification of DEGs. In the CG vs. T14 comparison (Figure 5D), intracellular cellular component (CC, GO:0005622), catalytic activity of molecular function (MF, GO:0003824), and metabolic process of biological process (BP, GO:0008152) were the dominant GO terms. In the CG vs. T7 comparison (Figure 5E), the most significant GO terms consisted of nuclear outer membrane–endoplasmic reticulum membrane network (CC, GO:0042175), catalytic activity (MF, GO:0003824), and monocarboxylic acid metabolic process (BP, GO:0032787). Moreover, DNA packaging complex (CC, GO:0044815), structural constituent of chromatin (MF, GO:0030527), and phosphorylation (BP, GO:0016310) were the top subcategories of GO terms in the T14 vs. T7 comparison (Figure 5F). For the KEGG enrichment analysis, herpes simplex virus 1 infection (ko05168), glycine, serine, and threonine metabolism (ko00260), and cysteine and methionine metabolism (ko00270) were the top three enriched pathways in the CG vs. T14 comparison (Figure 5G). Glycine, serine and threonine metabolism (ko00260), protein processing in the endoplasmic reticulum (ko04141), and steroid hormone biosynthesis (ko00140) were the most enriched pathways in the CG vs. T7 comparison (Figure 5H). Furthermore, DEGs in the T14 vs. T7 comparison (Figure 5I) were significantly clustered in cytokine–cytokine receptor interaction (ko04060), Toll-like receptor signaling pathway (ko04620), and cellular senescence (ko04218).

In addition, the DEGs of fatty acid biosynthesis (*Acsbg2, Acsl3, Acsl4, Acsl5, Mcat, and Acacb*), the biosynthesis of unsaturated fatty acids (*Hsd17b12*, *Elovl7*, *Scd*, and *Baat*), and steroid hormone biosynthesis (*Hsd17b12*, *Hsd17b3*, *Hsd17b2*, and *Dhrs11*) related to lipid metabolism were identified in both the CG vs. T14 and CG vs. T7 comparisons (Table S3). The DEGs in pyruvate metabolism (*Ldhb*, *Acat2*, and *Acacb*) and starch and sucrose metabolism (*Pgm1*, *Pgm2*, and *Treh*) were involved in carbohydrate metabolism; meanwhile, the DEGs in necroptosis (*Ticam1*, *Vdac1*, *Chmp2b*, *Ifnar2*, *Stat3*, *Slc25a5*, *Slc25a4*, *Hsp90aa1*, *Tnf*, *Glud1*, and *Tnfsf10*), the p53 signaling pathway (*Gadd45a*, *Siah1*, *Sesn2*, *Ccne2*, *Cdkn1a*, *Atr*, and *Tsc2*), and apoptosis (*Ddit3*, *Gadd45a*, *Lmnb1*, *Tuba1c*, *Tnf*, *Tnfsf10*, *Fos*, *Itpr1*, and *Ctso*) were associated with cell death.

To validate the reliability of the transcriptome analysis, ten genes in each pairwise comparison were randomly selected to perform the qRT-PCR experiment, including *Wnt*, *Hspa8*, *Hspa13*, *Hsph1*, *Tlr5*, *Tlr4*, *Tlr7*, *Tlr8*, *Dck*, and *Kmo* (Table S1, Figure S1). *Wnt*, involved in the Wnt signaling pathway, was up-regulated at the mRNA level in the T14 and T7 groups. The mRNA levels of *Hspa8*, *Hspa13*, and *Hsph1*, which were the key genes in protein processing in the endoplasmic reticulum, were increased in the T14 and T7 groups. *Tlr7* and *Tlr8*, enriched in the Toll-like receptor signaling pathway, were decreased at the transcription level in the T14 and T7 groups. The Pearson correlation coefficients were calculated between the RT-PCR and transcriptomic results. The R^2^ values of the Pearson correlation coefficients were 0.96, 0.97, and 0.98, corresponding to the comparison groups of CG vs. T14 (Figure S1B), CG vs. T7 (Figure S1D), and T14 vs. T7 (Figure S1F). These data indicate that the mRNA levels in the RT-PCR were consistent with the transcriptomic results, confirming the reliability and accuracy of the transcriptome analysis.

### 3.5. Effect of Cold Stress on Hepatic Metabolome

#### 3.5.1. Assessment of Metabolic Profiles

**Figure 6:**
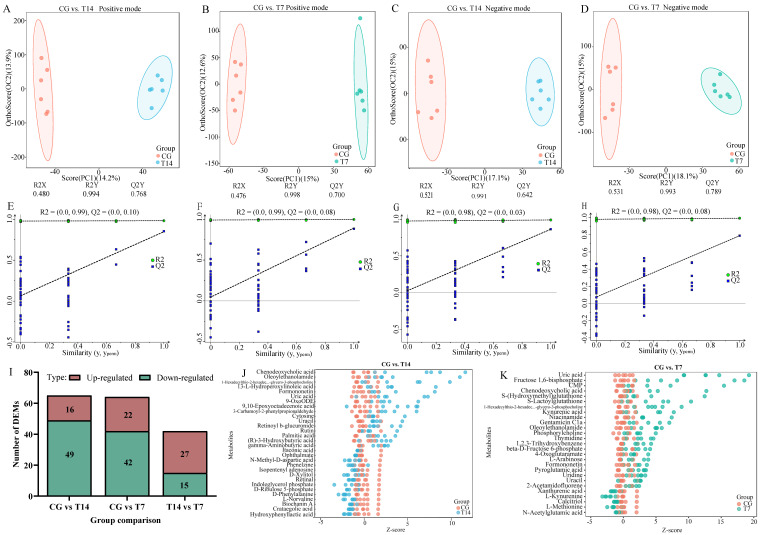
Multivariate statistical analysis and differentially expressed metabolites (DEMs) of the metabolome in different groups. Orthogonal projection to latent structures–discriminant analysis (OPLS-DA) score plots in positive mode (**A**,**B**) and negative mode (**C**,**D**). “R2Y” indicates the explanatory rate and “Q2Y” indicates the predictive ability of the OPLS-DA model. Permutation tests of the OPLS-DA models for the CG vs. T14 (**E**) and CG vs. T7 (**F**) comparisons in positive mode as well as the CG vs. T14 (**G**) and CG vs. T7 (**H**) comparisons in negative mode. (**I**) The number of DEMs in three pairwise comparisons. Z-score plots showing the top 30 DEMs for the CG vs. T14 (**J**) and CG vs. T7 (**K**) comparisons. “CG” indicates the control group. “T14” and “T7” indicate 14 °C and 7 °C cold stress groups.

The positive ion mode (POS) and negative ion mode (NEG) functions were utilized separately to improve the metabolite coverage and detection efficiency of the metabolome analysis. Multivariate statistical analyses, consisting of PLS-DA (Figure S2) and OPLS-DA (Figure 6A–H), were carried out to find the differences in metabolic profiles among the groups. The PLS-DA results showed that the metabolic profiles in the different groups could be separated in both the positive (Figure S2A,C,E) and negative modes (Figure S2B,D,F). Similarly, OPLS-DA showed a clear distinction between the T14 and T7 cold stress groups and the control group. R2Y and Q2, as the two parameters of cross-validation, were able to evaluate the stability and reliability of the OPLS-DA model. In the CG vs. T14 comparison, R2Y = 0.999 cum and Q2 = 0.768 cum in positive mode (Figure 6A,E), and R2Y = 0.991 cum and Q2 = 0.642 cum in negative mode (Figure 6C,G). In the CG vs. T7 comparison (Figure 6B,F), R2Y = 0.998 cum and Q2 = 0.700 cum in positive mode and R2Y = 0.993 cum and Q2 = 0.789 cum in negative mode (Figure 6D,H). The Y-intercept of Q2, as the parameter of the permutation tests, indicates the accuracy of the OPLS-DA model. For both the CG vs. T14 (Figure 6E,G) and CG vs. T7 (Figure 6F,H) comparisons, the Y-intercept of Q2 was lower than 0.2, suggesting the good accuracy of our OPLS-DA model.

#### 3.5.2. Identification and Enrichment Analysis of DEMs

As shown in Figure 6I, a total of 65 (16 up- and 49 down-regulated), 64 (22 up- and 42 down-regulated), and 42 (27 up- and 15 down-regulated) DEMs were detected in the CG vs. T14, CG vs. T7, and T14 vs. T7 pairwise comparisons, respectively. Furthermore, the Z-score plots exhibit the top DEMs in the pairwise comparisons (Figure 6J,K). Chenodeoxycholic acid, oleoylethanolamide, 1-hexadecylthio-2-hexadecanoylamino-1,2-dideoxy-sn-glycero-3-phosphocholine, 13-L-hydroperoxylinoleic acid, and formononetin were the top five DEMs in the CG vs. T14 (Figure 6J) comparison, while uric acid, fructose 1,6-bisphosphate, CMP, chenodeoxycholic acid, and S-(Hydroxymethyl)-glutathione were the most remarkable DEMs in the CG vs. T7 comparison (Figure 6K).

**Figure 7:**
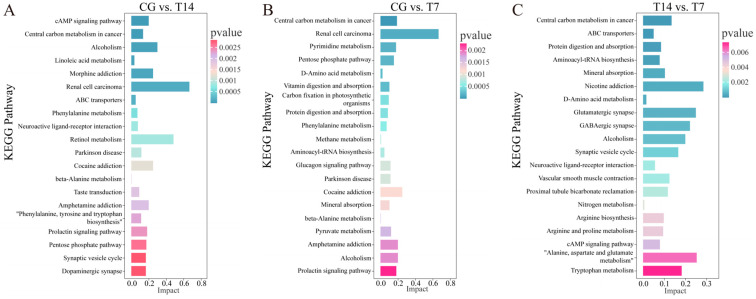
KEGG enrichment analysis of differentially expressed metabolites (DEMs) of the metabolome in three pairwise comparisons, including CG vs. T14 (**A**), CG vs. T7 (**B**), and T14 vs. T7 (**C**). “CG” indicates the control group. “T14” and “T7” indicate 14 °C and 7 °C cold stress groups.

The KEGG analyses of the DEMs (Figure 7) showed that the cAMP signaling pathway, central carbon metabolism in cancer, alcoholism, and linoleic acid metabolism were most significantly enriched in the CG vs. T14 (Figure 7A) comparison, while central carbon metabolism in cancer, renal cell carcinoma, pyrimidine metabolism, and the pentose phosphate pathway were the top significant pathways in the CG vs. T7 (Figure 7B) comparison. Different from the above comparisons, the predominant pathways of the DEMs in the T14 vs. T7 comparison comprised central carbon metabolism in cancer, ABC transporters, and protein digestion and absorption (Figure 7C).

### 3.6. Joint Analysis of DEGs and DEMs

**Figure 8:**
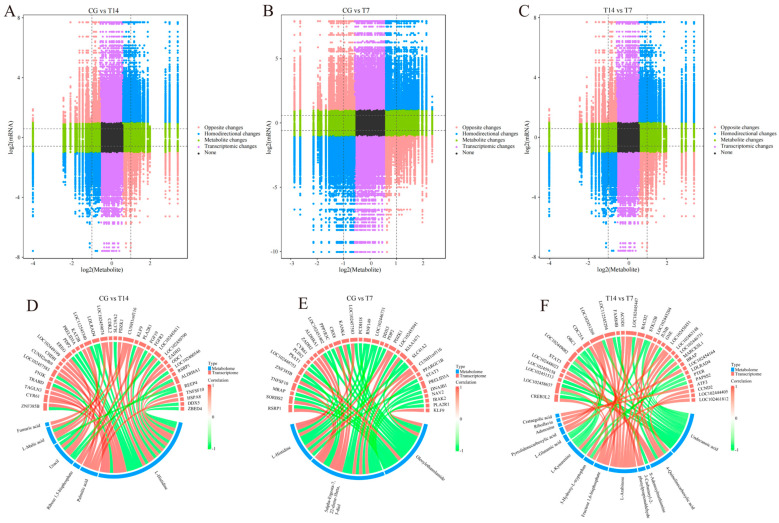
Nine-quadrant diagrams and chord diagrams showing the correlation of differentially expressed genes (DEGs) and metabolites (DEMs) in three comparisons. Nine-quadrant diagrams show the correlation of DEGs and DEMs in the CG vs. T14 (**A**), CG vs. T7 (**B**), and T14 vs. T7 (**C**) comparisons. The chord diagrams exhibit a significant association of DEGs and DEMs in the CG vs. T14 (**D**), CG vs. T7 (**E**), and T14 vs. T7 (**F**) comparisons. “CG” indicates the control group. “T14” and “T7” indicate 14 °C and 7 °C cold stress groups.

To establish the association between genes and metabolites and then further excavate the potential pathways involved in responding to cold stress, conjoint analyses were applied to the transcriptomic and metabolomic results. The variation in all DEMs and DEGs in each pairwise comparison was exhibited using nine-quadrant diagrams (Figure 8A–C), with Pearson |r| > 0.80 and *p* < 0.05. The DEMs and DEGs in quadrants 3 and 7 were positively associated, but they were negatively related in quadrants 1 and 9. The top DEMs strongly correlated with DEGs were further exhibited with correlation networks (Figure 8D–F). In the CG vs. T14 comparison (Figure 8D), L-histidine was positively associated with *Znf385b*, *Cyr61*, *Tagln3*, *Fn3k*, and *Chdh* genes, and negatively related to *Trabd*, *Ereg*, *Pdp2*, *Prelid3a*, and *Ldlrad4*. Palmitic acid was positively correlated with *Kat2b*, *Fgfr3*, *Odc1*, *Hspa8*, and *Ddx5*. Uracil was highly associated with *Trabd*, *Ereg*, *Klf9*, *Znf385b*, *Aldh8a1*, and *Rsrp1*. In the CG vs. T7 comparison (Figure 8E), L-histidine was positively related to *Znf385b*, *Cyr61*, and *Pdzk1*, as well as *Rsrp1,* and negatively associated with *Rnf149*, *Pdp2*, and *Slc41a2*, as well *Prelid3a*. Oleoylethanolamide was negatively correlated with *Rsrp1, Mrap, Tnfsf10, Psat1, and Aldh8a1*. In the T14 vs. T7 comparison (Figure 8F), undecanoic acid was negatively correlated with *Creb3l2*, *Stat3*, *Gne*, and *Stk32b,* while L-arabinose was positively related to *Junb*, *Marcksl1*, *Brap*, and *Tmem119*.

**Figure 9:**
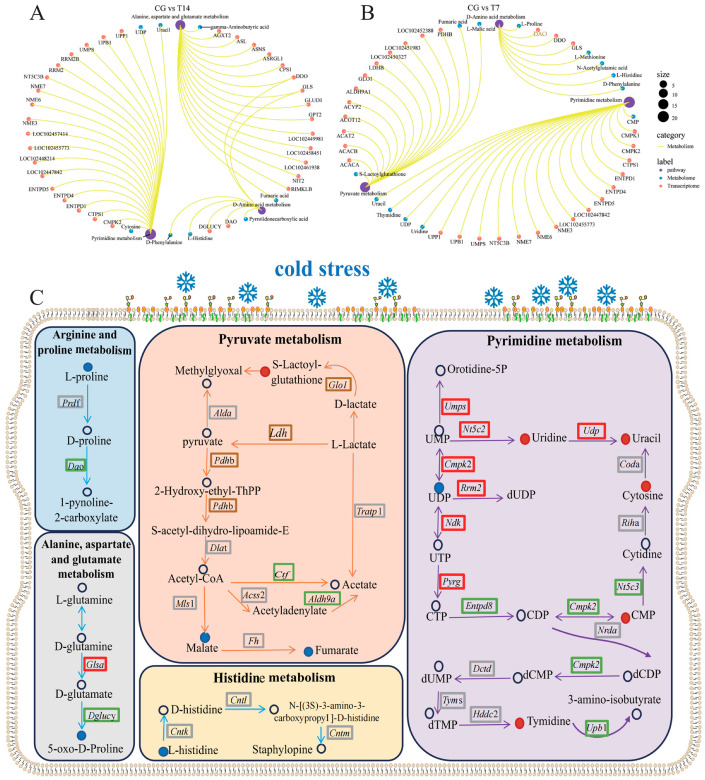
The enriched pathways in response to cold stress, assessed by conjoint analysis of differentially expressed genes (DEGs) and metabolites (DEMs). Enriched pathways of CG vs. T14 (**A**) and CG vs. T7 (**B**) comparisons. (**C**) The network maps illustrates the predominant genes, metabolites, and signaling pathways responding to cold stress. “CG” indicates the control group. “T14” and “T7” indicate 14 °C and 7 °C cold stress groups.

Compared with the control group, the DEGs and DEMs in the T14 group (Figure 9A,C) were commonly enriched in the three KEGG pathways, including pyrimidine metabolism, alanine, aspartate and glutamate metabolism, and D-amino acid metabolism. In the CG vs. T7 comparison (Figure 9B,C), pyrimidine metabolism, D-amino acid metabolism, and pyruvate metabolism were the most enriched KEGG pathways of the DEGs and DEMs. It is worth noting that D-amino acid metabolism and pyrimidine metabolism were the common KEGG pathways in both the CG vs. T14 and CG vs. T7 comparisons.

## 4. Discussion

Owing to the great damage to aquatic animals induced by the natural cold encountered outdoors, the responsive mechanisms of various animals, such as black porgy [13], largemouth bass [27], and kuruma shrimp (*Marsupenaeus japonicus*), to acute temperature change have been reported [28]. Cold stress may lead to growth retardation and high mortality, affecting the structural and functional integrity of tissues, metabolic processes, and physiological function [29]. Until now, the comprehensive effects of low temperature on the Chinese soft-shelled turtle have remained obscure. In the current study, plasma biochemical parameters, histological features, and cell apoptosis in the liver were analyzed to reveal the influence of cold stress on physiological processes and hepatic histopathological damage. Furthermore, joint analyses of the transcriptome and metabolism were conducted to reveal the metabolic process and molecular mechanism of the liver in response to cold stress in the Chinese soft-shelled turtle.

### 4.1. Energy Metabolism Responding to Cold Stress

Plasma GLU, TG, CHO, HDL, and LDL, as typical energy-related parameters, are considered to be biomarkers of physiological response to temperature stress [30]. Our research found that plasma GLU content rapidly decreased in the Chinese soft-shelled turtles exposed to 14 °C and 7 °C cold stress. Rapid reductions in blood glucose have been found in *Piaractus mesopotamicus* [31] and *Chanos chanos* under low-temperature stress [32]. Low-temperature stress may improve the energy expenditure of fish to deal with metabolic disorders, resulting in the reduction in GLU levels [33]. These decreases in blood glucose might indicate an adaptive capacity to rapidly switch towards other energy sources, such as lipid metabolism [34]. HDL and LDL are two lipoprotein members, transporting lipids between the blood and tissues. The function of LDL is primarily to transport lipids from the intestine and liver to the peripheral tissues; meanwhile, HDL is mainly involved in reverse cholesterol transport [35]. Our results showed that plasma LDL content increased from 1 to 2 dps and decreased to a lower level from 8 to 16 dps, whereas plasma HDL exhibited a converse trend to LDL. In addition, plasma TG and CHO were kept at lower levels after cold stress. These data might indicate that the TG and CHO produced by the liver are transported to the peripheral tissues by LDL to resist cold stress at the initial stage. Furthermore, plasma TG and CHO are important fuels that can supply energy for the Chinese soft-shelled turtle to keep a balanced state at low temperatures.

The liver, as the key organ involved in energy metabolism, is deemed to play an important part in maintaining the balance of energy substances in blood [36]. The present research identified abundant DEGs and DEMs associated with carbohydrate or lipid metabolism in the liver via transcriptome and metabolism analyses. For example, the genes in fructose and mannose metabolism (*Pfkfb4*, *Tigar*, and *Pmm2*) and starch and sucrose metabolism (*Pgm2* and *G6pc2*) were overexpressed, regulating the carbohydrate metabolism of the liver exposed to cold stress. In addition, the genes enriched in lipid metabolism, including fatty acid degradation (*Acsbg2*, *Acsl3*, *Acsl4*, and *Acsl5*), as well as the biosynthesis of unsaturated fatty acids (*Hsd17b12*, *Elovl7*, and *Scd*) and metabolites in linoleic acid metabolism (13-L-Hydroperoxylinoleic acid; 9-OxoODE; 9,10-Epoxyoctadecenoic acid; 9,10-DHOME), were increased under cold stress. In general, our data implied that both carbohydrate metabolism and lipid metabolism might be activated to respond to cold stress in the liver of the Chinese soft-shelled turtle.

### 4.2. Tissue Damage and Cellular Apoptosis of the Liver Caused by Cold Stress

Plasma ALT, AST, and AKP can be used to evaluate the health status of animals under acute stressors [37]. The elevation of plasma ALT and AST activities indicates liver damage [38]. Herein, the 14 °C and 7 °C low temperatures remarkably increased plasma ALT and AST activities after 2 dps, suggesting that liver tissue might be damaged after cold stress. This conclusion was verified with the subsequent liver histopathological results.

Histopathology is a key biomarker to assess the health status of organs or tissues under stress. For example, histological analysis has found that heat stress increases the densities of melano-macrophage and fragmented nuclear in the head kidney and spleen of the largemouth bass [27]. The histopathological images in this study showed that the liver tissue was damaged in the Chinese soft-shelled turtles exposed to 14 °C and 7 °C cold stress. Moreover, the injuries were further aggravated in the 7 °C stress group with the prolongation of stress time. In detail, the hepatic sinuses were enlarged and congested with erythrocytes, implying that the cold stress obstructed the circulatory system [39]. Obscure or dissolved hepatocyte boundaries, abnormal nuclei, including nuclear migration, nuclear deformation, and nuclear swelling, and even large-scale hepatocyte necrosis were also observed in the two cold stress groups. These injuries suggest that the low temperature destroyed the structure of the cellular membranes and nuclei of the liver. Similar damage has been observed in the liver tissue of black porgy exposed to low temperature [27]. In addition, the numbers of hemosiderin depositions were elevated after low-temperature stress in our research. Hemosiderin deposition, caused by an excess ion deposition in the liver, spleen, and bone marrow, is a typical symptom of hemosiderosis disease [40]. Hepatic hemosiderosis is related to several factors, such as excessive amounts of iron in the diet, endogenous stress, and the presence of chronic inflammatory processes [40]. Hepatic hemosiderosis has also been found in Chinese soft-shelled turtles with white abdominal disease [41]. In addition, our research showed that the amount of steatosis was increased in the 14 °C and 7 °C cold stress groups. This phenomenon may be due to low-temperature stress leading to metabolic disorders, and subsequently causing an excessive accumulation of lipids. Similar results have been found in the liver of *Seriola lalandi* [42] and black porgy [13] under temperature stress.

Previous studies have found that temperature stress could lead to cellular damage and apoptosis in animals [43,44]. In our study, the 7 °C cold stress triggered apoptosis in Chinese soft-shelled turtle liver, which was aggravated with the extension of cold stress time. The present transcriptome also revealed that the expression of genes in the p53 signaling pathway (*Gadd45a*, *Siah1*, *Ccnb2*, *Rrm2*, *Mdm2*, *Sesn2*, *Ccne2*, *Rrm2b*, and *Cdkn1a*) and in apoptosis (*Map3k5*, *Kras*, *Tuba1c*, *Ddit3*, *Mapk9*, *Gadd45a*, *Faslg*, and *Lmnb1*) was up-regulated. In general, these results indicate that the low temperature induced hepatocyte apoptosis by altering the gene expression involved in apoptosis; moreover, the accumulation of apoptosis might be related to histological damage to the liver in the Chinese soft-shelled turtle.

### 4.3. Key Signaling Pathways Responding to Cold Stress

#### 4.3.1. Pyrimidine Metabolism

Pyrimidine metabolism and purine metabolism are known as nucleotide metabolism, and have an important role in regulating multiple biochemical processes in cells, such as energy supply and metabolic pathways [45]. It has been reported that the disorder of pyrimidine metabolism under various stresses can cause DNA damage [46]. Herein, the levels of metabolites (Uridine, uracil, cytosine, CMP, and Tymidine) and gene expression (*Umps*, *Nt5c2*, *Udp*, *Cmpk2*, *Rrm2*, *Ndk*, and *Pyrg*), enriched in the pyrimidine metabolism, were regulated under cold stress. Previous studies have also found a role of pyrimidine metabolism in response to primary oxidative stress. For example, high temperature stress has been found to alter the pyrimidine metabolism in tsinling lenok trout [47]. This finding is similar to our results, meaning that cold stress can lead to severe DNA damage in the Chinese soft-shelled turtle, coinciding with the above apoptotic results.

#### 4.3.2. Amino Acid Metabolism and Pyruvate

Pyruvate metabolism is closely connected with the synthesis of glucose, glycerol, fatty acids, and non-essential amino acids via the pyruvate, which is a crucial intermediate involved in various metabolic pathways [48]. Therefore, pyruvate metabolism is a crucial pathway in regulating energy homeostasis. In addition, pyruvate metabolism also has an important part in immune function, affecting macrophage and T-cell immunity [49]. Fumarate is involved in multiple signaling pathways, including the citrate cycle of carbon metabolism and the oxidative phosphorylation of energy metabolism. The accumulation of fumarate can lead to oxidative stress in primary mouse kidney cells [50]. In our research, genes including *Glol*, *Ldh*, *Pdhb*, *Ctf*, and *Aldh9a,* and metabolites including S-lactoylglutathione, fumarate, and malate, enriched in pyruvate metabolism, were remarkably altered after cold stress. This result indicated that cold stress might affect energy metabolism or amino acid metabolism by altering pyruvate metabolism.

Amino acids are pools for protein synthesis and functioning. It has been reported that various stress-responding metabolites have been synthesized by the amino acid metabolism, suggesting that amino acid metabolism could play an important role in the response to stress in fish [51]. It has been reported that amino acid metabolism is regulated under low-temperature stress in various aquatic animals, including in *Puntius tetrazona* [52], kuruma shrimp [28], and yellow drum [12]. In this research, L-proline in the arginine and proline metabolism, 5-oxo-D-proline in the alanine, aspartate, and glutamate metabolism, and L-histidine in the histidine metabolism were decreased after cold stress. Similarly, the abundance of L-histidine significantly declined in the heat-stressed gills of *Alosa sapidissima* [53]. Proline might be involved in promoting fish to resist low-temperature stress [9]. Therefore, our data might hint that L-proline, 5-oxo-D-proline, and L-histidine in the amino acid metabolism were consumed to assist the Chinese soft-shelled turtle in coping with cold stress.

## 5. Conclusions

The current research found that the contents of plasma energy-related parameters were reduced in a time-dependent manner to cope with cold stress in Chinese soft-shelled turtles. The cold stress induced histological damage and cell apoptosis in liver tissue. Abundant DEGs (*Acsl3, Acsl4, Pgm1*, *Cdkn1a*, *Atr*, *Ddit3*, *Fos*, and *Itpr1*) and DEMs (chenodeoxycholic acid, linoleic acid, 6-Phosphogluconic acid, fumaric acid, and D-mannose), respectively excavated by comparative transcriptome and metabolism analyses, were clustered in the pathways of energy metabolism and cell death. The conjoint analysis of the two omics revealed that pyrimidine metabolism, amino acid metabolism, and pyruvate metabolism are the crucial pathways for the Chinese soft-shelled turtle to resist cold stress.

## Data Availability

All data generated and analyzed during this study are included in this published article. All raw RNA sequencing data have been submitted to the NCBI Sequence Read Archive (SRA) with the BioProject ID PRJNA1167896.

## Supplementary Materials

The following supporting information can be downloaded at: https://www.mdpi.com/article/10.3390/biology14010055/s1, Figure S1. Pearson correlation analysis of liver mRNA levels: real-time PCR and RNA-Seq results of CG vs. T14 (A,B), CG vs. T7 (C,D), and T14 vs. T7 comparisons (E,F). “CG” indicates the control group. “T14” and “T7” indicate 14 °C and 7 °C cold stress groups. Figure S2. Projection to latent structures–discriminant analysis (PLS-DA) of the metabolome in different groups. PLS-DA score plots of CG vs. T14 (A,B), CG vs. T7 (C,D), and T14 vs. T7 comparisons (E,F) in both positive and negative modes. “CG” indicates the control group. “T14” and “T7” indicate 14 °C and 7 °C cold stress groups. Table S1. The qRT-PCR primer sequences for Pelodiscus Sinensis. Table S2. Overview of the sequencing quality of the transcriptome. Table S3. Fifferentially expressed genes and pathways enriched in energy metabolism and cell death. Table S4. Differentially expressed metabolites and pathways involved in energy metabolism and cell death.
