# Supplementary material for: Biochemical, Histological, and Multi-Omics Analyses Reveal the Molecular and Metabolic Mechanisms of Cold Stress Response in the Chinese Soft-Shelled Turtle (Pelodiscus sinensis)
Source: Biology (Basel). 2025 Jan 11;14(1):55. doi: 10.3390/biology14010055 (PMC11760877; doi:10.3390/biology14010055)
Supplement: Supplementary file 1 [file biology-14-00055-s001.zip › Supplementary figures-edited.pdf]

Supplementary figure

Fig. S1

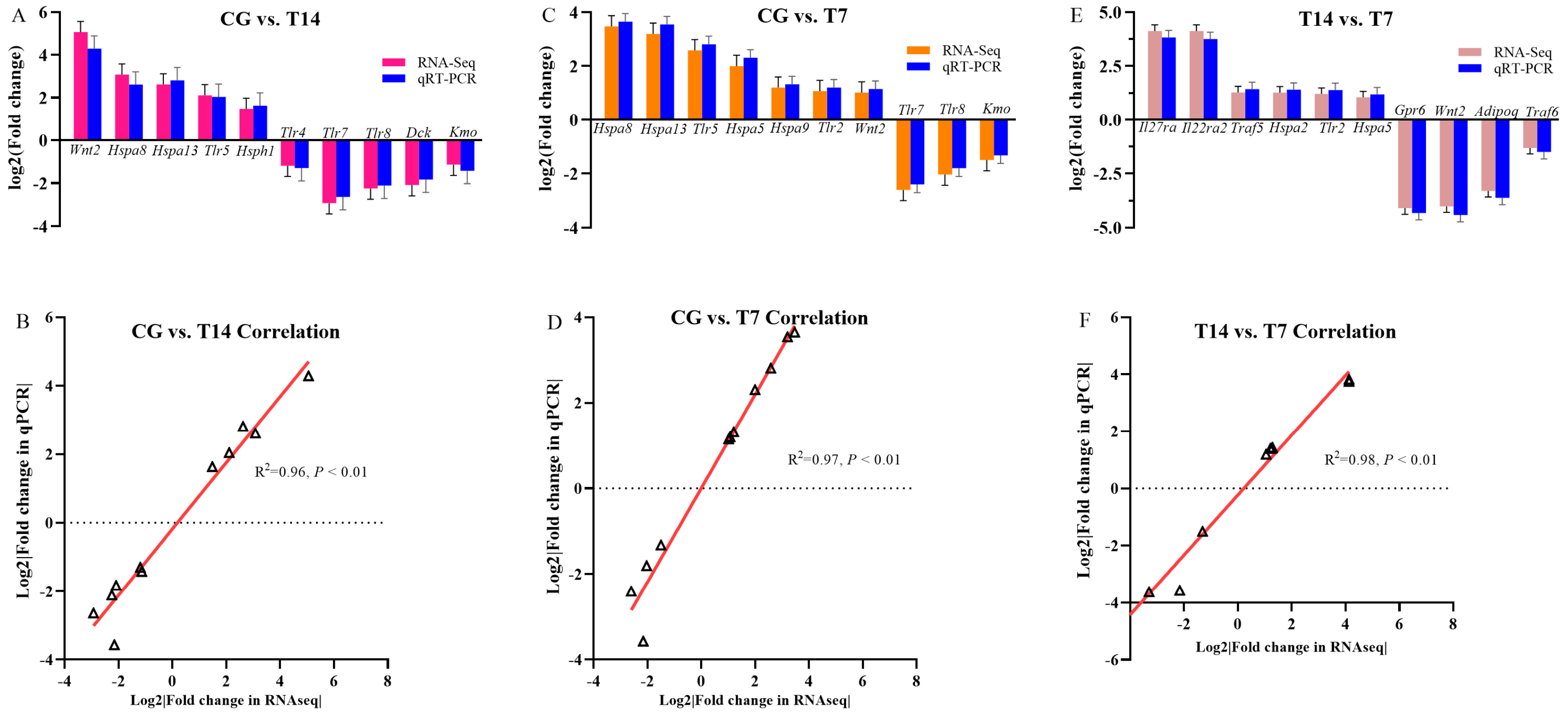

Fig. S1. Pearson correlation analysis of liver mRNA levels: real-time PCR and RNA-Seq results of CG vs. T14 (A-B), CG vs. T7 (C-D), and T14 vs. T7 comparisons (E-F). “CG” indicates the control group. “T14” and “T7” indicate 14 °C and 7 °C cold stress groups.

Fig. S2

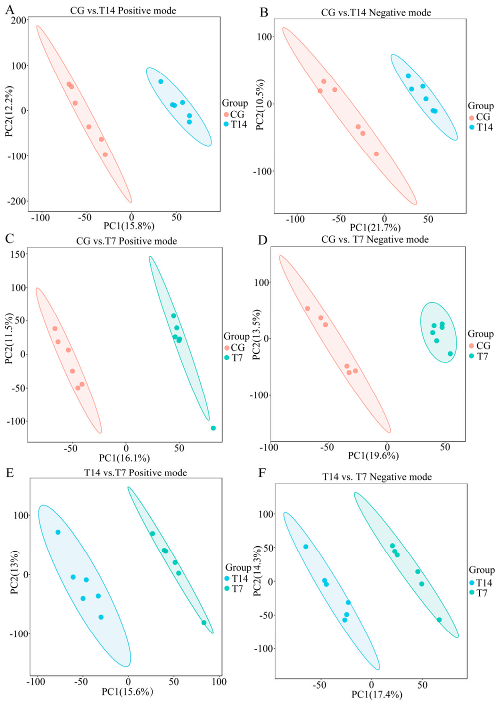

Fig. S2. Projection to latent structures–discriminant analysis (PLS-DA) of the metabolome in different groups. PLS-DA score plots of CG vs. T14 (A-B), CG vs. T7 (C-D), and T14 vs. T7 comparisons (E-F) in both positive and negative modes. “CG” indicates the control group. “T14” and “T7” indicate 14 °C and 7 °C cold stress groups.
