# Supplementary material for: Biochemical, Histological, and Multi-Omics Analyses Reveal the Molecular and Metabolic Mechanisms of Cold Stress Response in the Chinese Soft-Shelled Turtle (Pelodiscus sinensis)
Source: Biology (Basel). 2025 Jan 11;14(1):55. doi: 10.3390/biology14010055 (PMC11760877; doi:10.3390/biology14010055)
Supplement: Supplementary file 1 [file biology-14-00055-s001.zip › Supplementary tables-edited.pdf]

Supplementary Tables

Table S1. The qRT-PCR primer sequences for *Pelodiscus Sinensis*

Gene symbol	Gene name	Forward primer (5'-3')	Reverse primer (5'-3')	NCBI Accession number	Product length (bp)
Wnt2	Wnt family member 2	CAAGACGGCACTGGTTTCAC	GTCCCAAGGGAGCCTACATC	XM_006122543.3	125
Hspa2	heat shock protein family A (Hsp70) member 2	TCTTGAGTCCTACACCTA	CACTTTCTGCTTGTCTTG	NM_001287561.1	88
Hspa5	heat shock protein family A (Hsp70) member 5	GTAACAATCAAGGTCTAT	ATTCCATTCACATCTATC	NM_001286892.1	143
Hspa8	heat shock protein family A (Hsp70) member 8	CGTTGCCTTCACAGATAC	CGTGTTGGTAGGATTCATT	NM_001286908.1	76
Hspa9	heat shock protein family A (Hsp70) member 9	AATACATTCTACGCTACC	CTTCATCTTCATCAATACAA	XM_014569866.2	183
Hspa13	heat shock protein family A (Hsp70) member 13	ACTCTACACCATTCTTCTC	TTCAACACCTGCTCAATA	XM_006136928.3	275
Tlr2	toll-like receptor 2	TACAGATGCAAGCATTAAC	CAAACTACTAAGATCTGAAAAT	XM_025185298.1	205
Tlr5	toll-like receptor 5	TCTCACTGTTCATCTTCA	TATCTATTGCTTGCTTACG	XM_006115600	144
Hsph1	heat shock protein family H (Hsp110) member 1	GAATGGATGAGTAATGCTA	ATTGTGTAGTTCCTTGAG	XM_006125320.1	105
Tlr4	toll-like receptor 4	TGCTGGCATTCTGTTCGTG	GGATGATGTTGGTAGTGATGGG	NM_001286933	257
Tlr7	toll-like receptor 7	CTTGTCAGGTAATGCTAT	TGTTATCACTCAGGTCTA	XM_014573459.2	161
Tlr8	toll-like receptor 8	CAGCATAGTGTTCTCTTG	ACCGTAGTTCATTCATTAC	XM_014573456	277
Dck	deoxycytidine kinase	GGCATACACTTTCCAGAC	GCTCGAAGAAGACCACA	XM_025178440	107
Kmo	kynurenine 3-monooxygenase isoform X1	TGAACGCTGGATTTGAA	TGGCGAGGTCGGAGATA	XM_006113340	132
Il27ra	interleukin 27 receptor subunit alpha	CTCGCCCACTACACCCTCTA	ACTTGGCTCCTGCCTTCACC	XM_025180059.1	197
Il22ra2	interleukin 22 receptor subunit alpha 2	GTCCATTCCGATTGGAACAC	CATTGGTATCTTGCTGCCTC	XM_025178099.1	162
Traf5	TNF receptor associated factor 5	CGTCCACATCAGTATTCA	TTCTTCATCTTGTAGTCCAT	XM_006131089.3	120
Traf6	TNF receptor associated factor 6	CTTCACAGAGGAGATTCAA	GTAGAGCCATCAAACAGA	XM_006124469.3	89
Gpr6	G protein-coupled receptor 6	TCCCGATCAGCACGAAC	CACTTCTGCCTGCTACTT	XM_006123249.3	176
Adipoq	adiponectin, C1Q and collagen domain containing	ACTACGATGAAACCACAGGG	GCACTCAGGTGAAGCAAAAC	XM_006125187.3	196
β-actin	beta-actin	AGACCCGACAGACTACCTCA	CACCTGACCATCAGGCAACT	XM_006134860.1	193

Table S2. Overview of the sequencing quality of the transcriptome.

Sample	Raw Reads	CR	CR Ratio (%)	Q20 (%)	Q30 (%)	GC Content (%)	TMG (%)
CG1	43870158	43267862	98.63	98.5	95.73	50.37	86.8
CG2	44721662	44094936	98.6	98.52	95.75	50.43	88.47
CG3	43635300	43052128	98.66	98.61	96.05	49.95	89.22
T14_1	45969782	45330434	98.61	98.5	95.72	50.23	88.8
T14_2	44809818	44218720	98.68	98.57	95.93	50.12	89.72
T14_3	37807254	37330744	98.74	98.52	95.72	50.46	89.02
T7_1	44438030	43850380	98.68	98.49	95.63	49.87	89.04
T7_2	45559604	44992526	98.76	98.6	95.98	50.19	87.95
T7_3	42474054	41880488	98.6	98.56	95.91	49.99	87.76

For convenience, “CG” indicates the control group. “T14” and “T7” indicate 14 ℃ and 7 ℃ cold stress groups. “CR” indicates clean reads. “TMG” indicates the ratio of the total clean reads mapped to the genome.

Table S3. Fifferentially expressed genes and pathways enriched in energy metabolism and cell death.

CG vs T14			CG vs T7		
Pathway	Level 2 pathway	DEGs	Pathway	Level 2 pathway	DEGs
Primary bile acid biosynthesis	Lipid metabolism	*LOC102449598; Acox2; LOC102458362; Baat; LOC102457003; LOC102461871; Ch25h; LOC102462148*	Primary bile acid biosynthesis	Lipid metabolism	*LOC102457003; LOC102461871; Ch25h; LOC102449598; LOC102458362; Baat*
Steroid hormone biosynthesis	Lipid metabolism	*Hsd17b12; Srd5a1; Hsd17b3; Hsd17b2; LOC102460451; LOC102462176; Dhrs11; Comt; LOC102443979; LOC106731311; LOC102457003; LOC102460158; LOC102459148; LOC102462148; LOC102444110; LOC102445634*	Steroid hormone biosynthesis	Lipid metabolism	*Hsd17b3; Hsd17b12; LOC102457003; LOC102462423; LOC102445634; LOC102462176; LOC102459465; LOC102446253; LOC102444110; LOC102446483; Hsd17b2; LOC102443979; LOC112544543; LOC102444001; LOC102459148; Dhrs11*
Fatty acid biosynthesis	Lipid metabolism	*Acsl5; Htd2; Acsl4; Acsbg2; Acsl3; Mcat; Acacb*	Fatty acid biosynthesis	Lipid metabolism	*Acsbg2; Acsl5; Acsl4; Acsl3; Mcat; Acaca; Acacb*
Fatty acid degradation	Lipid metabolism	*Acsl4; Acsl3; Acsbg2; Acsl5; LOC102457687; LOC102452388; Aldh9a1; Acat2; Aldh2; Eci2; Acaa2*	Fatty acid degradation	Lipid metabolism	*Acsbg2; LOC102457687; Acsl3; Acsl4; Acsl5; Acat2; LOC102451983; Acox1; Eci2; Echs1; Aldh9a1; LOC102452388*
alpha-Linolenic acid metabolism	Lipid metabolism	*LOC102450450; Fads2; LOC106732455; LOC102443502; LOC102459717; LOC106732453; LOC102459481*	alpha-Linolenic acid metabolism	Lipid metabolism	*LOC106732453; LOC102450450; Plb1; LOC102462875; LOC102459481; Acox1; LOC102443502*
Biosynthesis of unsaturated fatty acids	Lipid metabolism	*Hsd17b12; Elovl7; Scd; LOC102447684; Fads2; LOC102447939; Baat; LOC102461871*	Biosynthesis of unsaturated fatty acids	Lipid metabolism	*Scd; Hsd17b12; Elovl7; Scd5; LOC102461871; LOC102459125; Acox1; LOC112544543; Baat*
Glycerolipid metabolism	Lipid metabolism	*Dgkq; Lpl; Pnpla2; LOC102443833; Agpat2; LOC102443676; Aldh9a1; LOC102449336; Aldh2; Plpp3; Mgll; Dgka; Glyctk*	Linoleic acid metabolism	Lipid metabolism	*LOC106732453; LOC102450450; Plb1; LOC102462875; LOC102449417; LOC102443502; LOC102459481*
Amino sugar and nucleotide sugar metabolism	Carbohydrate metabolism	*LOC102462140; Gnpda2; Pgm2; Pmm2; LOC102447192; Uap1; Uxs1; LOC102447870; Npl; LOC102450356; Gne; Pgm1; Hexb*	Amino sugar and nucleotide sugar metabolism	Carbohydrate metabolism	*Pgm2; Pmm2; LOC102462140; LOC102447192; Uap1; Hexb; LOC102451581; Pgm1; Hk1; LOC102450356*

Pyruvate metabolism	Carbohydrate metabolism	*Ldhb; Aldh2; Acat2; LOC102452388; Me1; Aldh9a1; Acot12; LOC102450327; Acacb; LOC106731579*	Pyruvate metabolism	Carbohydrate metabolism	*Ldhb; Glo1; Pdhbacot12; LOC102452388; Acyp2; Aldh9a1; Acacb; Acaca; LOC102450327; Acat2; LOC102451983*
Fructose and mannose metabolism	Carbohydrate metabolism	*Pmm2; Pfkfb4; Tigar; LOC102447192; Enosf1; LOC102447388; LOC102445123; Sord*	Fructose and mannose metabolism	Carbohydrate metabolism	*Pfkfb4; LOC102447192; Tigar; Pmm2; Sord; LOC102451581; LOC102445123; Hk1; Enosf1*
Starch and sucrose metabolism	Carbohydrate metabolism	*Pgm2; Pygm; LOC102447192; Amy2a; LOC102447842; Pgm1; LOC102462946; LOC102451082; Treh*	Starch and sucrose metabolism	Carbohydrate metabolism	*Pgm2; G6pc2; LOC102447192; LOC102451581; Pgm1; Si; LOC102447842; Treh; LOC102451082; Hk1*
Butanoate metabolism	Carbohydrate metabolism	*Hmgcs1; LOC102446448; LOC102445731; LOC102450712; LOC102445968; Acat2*	Butanoate metabolism	Carbohydrate metabolism	*Hmgcs1; LOC102450712; LOC102446448; LOC102445968; Echs1; Acat2*
Ascorbate and aldarate metabolism	Carbohydrate metabolism	*LOC102460451; Aldh9a1; LOC102443979; Aldh2; Kl; LOC102453557*	Glycolysis / Gluconeogenesis	Carbohydrate metabolism	*LOC102447192; G6pc2; Pdhb; Pgm2; Ldhb; Hk1; LOC102451983; LOC102452388; LOC102451581; LOC102445123; Pgm1; Eno2; Aldh9a1*
Necroptosis	Cell growth and death	*Faslg; Ticam1; Mapk9; Dnm1l; Vdac1; Chmp2b; Ifnar2; Stat3; Slc25a5; Vps4b; Hmgb1; Pygm; Slc25a4; Hsp90aa1; Stat1; Tnf; Glud1; LOC102451285; Capn1; Eif2ak2; LOC102462142; Chmp6; Tnfsf10; Zbp1; Tlr4*	Necroptosis	Cell growth and death	*Chmp2b; Mlkl; LOC102453347; Fas; Slc25a5; LOC102462633; Stat3; Hsp90aa1; Ifnar2; Slc25a6; Ticam2; Vdac1; LOC102451028; Slc25a4; LOC102447009; Ticam1; Tnfsf10;Glud1; Tnf*
Ferroptosis	Cell growth and death	*Hmox1; Acsl3; Slc39a14; Map1lc3b; Cp; Acsl4; Lpcat3; Acsl5; Slc40a1*	Ferroptosis	Cell growth and death	*Hmox1; Map1lc3b; Cp; Lpcat3; Tf; Acsl5; Acsl3; Acsl4; Slc39a14; Slc40a1; LOC102452940*
p53 signaling pathway	Cell growth and death	*Gadd45a; LOC102455941; Siah1; Ccnb2; Rrm2; Mdm2; Sesn2; LOC102454517; Ccne2; LOC102462633; Rrm2b; Cdkn1a; Atr; Cd82; Zmat3; Pidd1; Tsc2*	p53 signaling pathway	Cell growth and death	*Cdkn1a; Ei24; Gadd45a; LOC102455941; LOC102462633; Fas; Ccne2; Sesn2; Igf1; Tsc2; Ccnd2; Perp; Atr*
Apoptosis	Cell growth and death	*Map3k5; Kras; LOC102450295; LOC102462633; LOC102463731; Tuba1c; LOC102457516; Ddit3; LOC102457342; LOC102457222; Mapk9; Gadd45a; Faslg; LOC102448753; Lmnb1; LOC102462304; LOC102455941; LOC102461598; LOC102462482; LOC102454736; Tnf; Tnfsf10; Pidd1; Capn1; LOC102458936; Ikbkg; Dab2ip; Fos; LOC106732727; Ctso; Diablo; LOC102463497; LOC102446591; Itpr1; LOC102459334*	Apoptosis	Cell growth and death	*Ddit3; Akt3; Fas; LOC102457222; LOC102448753; LOC106731510; LOC102457342; LOC102462633; LOC102455941; Gadd45a; Lmnb1; LOC102458214; LOC102462304; Tuba1c; Tnfsf10; LOC102463497; Hras; Fos; LOC102462482; Itpr1; LOC102454736; Tnf; LOC102459436; Ctso*

Table S4. Differentially expressed metabolites and pathways involved in energy metabolism and cell death.

CG vs T14			CG vs T7		
Pathway	Level 2 pathway	DEMs	Pathway	Level 2 pathway	DEMs
Linoleic acid metabolism	Lipid metabolism	13-L-Hydroperoxylinoleic acid; 9-OxoODE; 9,10-Epoxyoctadecenoic acid; 9,10-DHOME	Linoleic acid metabolism	Lipid metabolism	Linoleic acid
Secondary bile acid biosynthesis	Lipid metabolism	Chenodeoxycholic acid	Secondary bile acid biosynthesis	Lipid metabolism	Chenodeoxycholic acid
Primary bile acid biosynthesis	Lipid metabolism	Chenodeoxycholic acid	Primary bile acid biosynthesis	Lipid metabolism	Chenodeoxycholic acid
Biosynthesis of unsaturated fatty acids	Lipid metabolism	Palmitic acid	Biosynthesis of unsaturated fatty acids	Lipid metabolism	Linoleic acid
Steroid hormone biosynthesis	Lipid metabolism	17alpha,21-Dihydroxypregnenolone	Steroid hormone biosynthesis	Lipid metabolism	17alpha,21-Dihydroxypregnenolone
Arachidonic acid metabolism	Lipid metabolism	19(R)-HETE	Arachidonic acid metabolism	Lipid metabolism	Prostaglandin H2; 19(R)-HETE
Pentose phosphate pathway	Carbohydrate metabolism	D-Ribulose 5-phosphate; 6-Phosphogluconic acid; Ribose 1,5-bisphosphate	Pentose phosphate pathway	Carbohydrate metabolism	D-Ribulose 5-phosphate; 6-Phosphogluconic acid; Fructose 1,6-bisphosphate; Sedoheptulose
Butanoate metabolism	Carbohydrate metabolism	Fumaric acid; gamma-Aminobutyric acid; (R)-3-Hydroxybutyric acid	Butanoate metabolism	Carbohydrate metabolism	Fumaric acid; 4-Hydroxybutanoic acid
Citrate cycle (TCA cycle)	Carbohydrate metabolism	Fumaric acid; L-Malic acid	Citrate cycle (TCA cycle)	Carbohydrate metabolism	Fumaric acid; L-Malic acid
Pyruvate metabolism	Carbohydrate metabolism	Fumaric acid; L-Malic acid	Pyruvate metabolism	Carbohydrate metabolism	Fumaric acid; L-Malic acid; S-Lactoylglutathione
Pentose and glucuronate interconversions	Carbohydrate metabolism	D-Ribulose 5-phosphate; D-Xylitol	Pentose and glucuronate interconversions	Carbohydrate metabolism	D-Ribulose 5-phosphate; L-Arabinose; D-Xylitol
Fructose and mannose metabolism	Carbohydrate metabolism	D-Mannose	Fructose and mannose metabolism	Carbohydrate metabolism	Fructose 1,6-bisphosphate

Glyoxylate and dicarboxylate metabolism	Carbohydrate metabolism	L-Malic acid	Glyoxylate and dicarboxylate metabolism	Carbohydrate metabolism	L-Malic acid
Amino sugar and nucleotide sugar metabolism	Carbohydrate metabolism	D-Mannose	Amino sugar and nucleotide sugar metabolism	Carbohydrate metabolism	L-Arabinose
Galactose metabolism	Carbohydrate metabolism	D-Mannose	Glycolysis / Gluconeogenesis	Carbohydrate metabolism	Fructose 1,6-bisphosphate
